# Aging-related impairment of neurogenic chloride secretion in human colon mucosa

**DOI:** 10.3389/fphys.2025.1540465

**Published:** 2025-03-19

**Authors:** Ruiyun Wang, Jing He, Pengcheng Yang, Tao Bai, Jun Song, Xiaohua Hou, Lei Zhang

**Affiliations:** ^1^ Department of Gerontology, Union Hospital, Tongji Medical College, Huazhong University of Science and Technology, Wuhan, China; ^2^ Department of Gastroenterology, Union Hospital, Tongji Medical College, Huazhong University of Science and Technology, Wuhan, China

**Keywords:** aging, intestinal epithelium, chloride secretion, elderly constipation, enteric neurodegeneration

## Abstract

**Background:**

lderly individuals are more susceptible to chronic constipation, which may be linked to imbalanced mucosa secretion and absorption. Our research aims to explore the age-related alterations in epithelial chloride secretion within the human colon.

**Methods:**

Colonic mucosal tissues were obtained from 9 young patients (aged 28–35 years), 10 middle-aged patients (aged 48–56 years), 10 elderly patients without constipation (aged 66–75 years), and 12 elderly patients with constipation (aged 65–78 years) who underwent surgery for colonic carcinoma. The epithelial chloride (Cl^−^) secretion was assessed using the short-circuit current (Isc) method. Comparative analysis was conducted on Cl^−^ secretion induced by spontaneous activity, bethanechol, forskolin, veratridine, and electrical field stimulation (EFS) in the four groups. Additionally, investigations were carried out on changes in cholinergic and VIPergic Cl^−^ secretion.

**Results:**

The spontaneous Cl^−^ secretion was not affected by aging. The increase in Isc induced by bethanechol and forskolin remained unaltered in aged colon. However, the veratridine-induced neurogenic Isc increment were significantly reduced with aging and constipation. The EFS-evoked Isc rising, which typically exhibiting a biphasic pattern, was inhibited by aging in a frequency-dependent manner. Administration of scopolamine and VIP_6-28_ to block cholinergic and vasoactive intestinal peptide (VIP) receptors led to smaller increases in the first and second phases of the EFS-evoked response in aged colons compared to young colons.

**Conclusion:**

Significant impairments in neurogenic Cl^−^ secretion occur in the aged colon, correlating with the degeneration of cholinergic and VIPergic nerves in the mucosa. This study could enhance our understanding of the pathophysiology of elderly constipation.

## 1 Introduction

Aging results in a progressive deterioration in physiological functions (Tran and Greenwood-Van Meerveld, 2014), impacting various aspects of gastrointestinal health. This decline manifests in gut dysbiosis (O'Toole and Jeffery, 2015), enteric neurodegeneration (Becker et al., 2018), reduced food intake (Kaipainen et al., 2022), impaired mucosal defense (Sato et al., 2015), and alterations in motility and sensation (Bitar et al., 2011; Keating et al., 2016) within the gastrointestinal tract. Consequently, there is a higher prevalence of gastrointestinal disorders in the elderly, with chronic constipation being a significant concern that affects their quality of life (Wiskur and Greenwood-Van Meerveld, 2010; Chu et al., 2014).

Elderly constipation is a challenging disorder with uncertain underlying mechanisms. For some individuals, issues such as colonic motility dysfunction and delayed colonic transit could contribute to the disorder (Wiskur and Greenwood-Van Meerveld, 2010; Whitehead et al., 2016). Age-related changes in the degenerative pathology of the myenteric plexus, along with an increased sensory threshold for rectal distension, impair normal defecation patterns and further complicate the issue (Tran and Greenwood-Van Meerveld, 2014; Keating et al., 2016; Rao et al., 2022; Vollebregt et al., 2021). Furthermore, maintaining a healthy balance of fluid secretion and absorption plays a crucial role in regular defecation (Krueger et al., 2016). However, the potential changes in intestinal epithelial secretion within the aged colon and their implications for elderly constipation remain unclear.

Chloride (Cl^−^) secretion is the predominant factor driving epithelial fluid secretion, influencing the volume of luminal fluid and playing a crucial role in conditions like diarrhea and constipation (Basavappa et al., 2005). Various Cl^−^ channels located on the apical membrane of enterocytes, such as cyclic adenosine monophosphate (cAMP)-dependent cystic fibrosis transmembrane regulator (CFTR) and Ca^2+^-activated chloride channel (CaCC), are potential therapeutic targets for these disorders (Krueger et al., 2016; Basavappa et al., 2005; Busby et al., 2010; Lunsford and Harris, 2010). The two channels, activated by specific agonists like forskolin (Fsk) and acetylcholine (ACh), facilitate basic and intensive secretion of Cl^−^ (Reynolds et al., 2007). However, the effect of aging on basal and induced Cl^−^ secretion via CFTR and CaCC remains to be definitively established.

Neurogenic mechanisms also play a pivotal role in the regulation of epithelial Cl^−^ secretion (Yang et al., 2014; Krueger et al., 2013). Secretomotor neurons within the submucosal plexus act on intestinal epithelial cells by releasing both secretion-promoting and secretion-inhibiting transmitters (Krueger et al., 2016; Baldassano et al., 2012). Mucosal nerve fibers are responsible for maintaining the basal secretion tone and regulating both spontaneous and nerve-evoked Cl^−^ secretion (Krueger et al., 2016). Cholinergic innervations are widespread throughout the gastrointestinal tract, with acetylcholine (ACh) acting as the primary mediator for nerve-evoked and baseline secretion in the intestine (Krueger et al., 2013; Bernard et al., 2009). Moreover, vasoactive intestinal polypeptide (VIP), another important secretion-promoting transmitter, has been found to raise intracellular cAMP levels and activate the CFTR channel in the colon (Baldassano et al., 2012; Fung et al., 2014; Traserra et al., 2023). The recent identification of aging-related enteric neurodegeneration highlights the loss of neurons and neural dysfunction (Wiskur and Greenwood-Van Meerveld, 2010; Wade and Cowen, 2004). Further exploration is required to elucidate the mechanisms underlying nerve-controlled epithelial secretion within aged colon.

This study focuses on assessing the impact of aging on epithelial Cl^−^ secretion in human colon mucosa through the measurement of short-circuit current (Isc). It may further enrich our knowledge in the pathophysiology of elderly constipation.

## 2 Materials and methods

### 2.1 Tissue collection and preparation

A total of 9 young patients (Y, median age was 31 years old), 10 middle aged patients (M, median age was 54 years old), 10 elderly patients without constipation (O-NC, median age was 71 years old) and 12 elderly patients with constipation (O-CC, median age was 70 years old) who underwent surgery of proximal colon that diagnosed with colonic carcinoma, terminal ileal stromal tumor or arteriovenous malformation at Wuhan Union Hospital were involved in this study. The elderly constipation should meet diagnostic criteria: (1) the disease course is at least 6 months, with symptoms present in the past 3 months; (2) spontaneous bowel movement <3 times per week; (3) more than 1/4 of defecation are dry ball feces or hard feces (Bristol Scale type 1 and 2). The exclusion criteria: (1) mechanical intestinal obstruction; (2) megacolon or pseudo obstruction; (3) intestinal inflammatory diseases; (4) with a history of gastrointestinal or abdominal surgery; (5) constipation caused by secondary causes, such as Parkinson disease, hypothyroidism, diabetes, depression, etc.,; (6) constipation caused by drugs and opioids; (7) use of anticholinergic drugs, antispasmodics, secretagogues, other gastrointestinal prokinetic drugs within 1 week; (8) serious cardiovascular, respiratory, liver, urinary and nervous system diseases, or systemic diseases and immunodeficiency diseases. The baseline information including age, gender, body mass index, disease course, presenting comorbidity, medication history, as well as the blood convention and biochemical indicators, were recorded and assessed. The key characteristic of included patients could be seen in Table 1. Mucosal tissue samples of the proximal colon were dissected from macroscopically unaffected areas >10 cm away from the lesion and immediately placed in ice cold Kreb’s solution oxygenated with 95% O_2_ + 5% CO_2_. All dissection of tissues was conducted in the ice-cold oxygenated Kreb’s solution. This study was approved by the Institutional Ethical Review Committee of the Huazhong University of Science and Technology, China, and all participants had signed informed consent ([2016] No.0728).

**TABLE 1:** Patient characteristics.

	Y (N = 9)	M (N = 10)	O-NC (N = 10)	O-CC (N = 12)
Age (years)	31 (28–35)	54 (48–56)	71 (66–75)	70 (65–78)
Gender				
Male	5	5	6	5
Female	4	5	4	7
Reasons for surgery				
Colonic carcinoma	6	8	10	10
Intestinal stromal tumors	2	2	0	1
Intestinal arteriovenous malformation	1	0	0	1
Presenting Comorbidity				
Chronic constipation	0	0	0	12
Chronic diarrhea	0	0	0	0
Diabetes	0	0	2	2
Parkinson disease	0	0	0	0
Hypothyroidism	0	0	0	0
Hypertension	0	1	3	5

Notes: Y, young; M, middle aged; O-NC, old without constipation; O-CC, old with chronic constipation.

### 2.2 Hematoxylin-eosin staining and histological assessment

The colonic tissues were fixed with 4% paraformaldehyde, embedded in paraffin and cut into slice. After routinely dewaxing and hydration, hematoxylin-eosin (HE) staining was conducted and then examined under a microscope (CX-31, OLYMPUS). The thickness of mucosal layer was measured at multiple points of each intestinal canal and the result was expressed as mean mucosal thickness (μm). The mucosal lymphocytes and goblet cells were counted in 10 contiguous non-overlapping fields per slide (magnification 400×) and the results were expressed as mean numbers of cells per high power field (numbers/hpf). The measurements and quantifications were carried out with the ImageJ software by the same investigator without knowledge of groupings.

### 2.3 Immunofluorescence analysis of enteric cholinergic and VIPergic fibers

The paraffin sections of colonic tissues were dewaxing, hydration and antigen repairing, then blocked with 5% bovine serum albumin for 1 h at room temperature. Slices were incubated with primary antibodies, anti-choline acetyltransferase (ChAT) (ab18736, Abcam) and anti-VIP (ab8556, Abcam), overnight at 4°C respectively, and then stained with Alexa Fluor 488 (green) or 594 (red) conjugated secondary antibodies accordingly. The nuclei were stained with DAPI (1 μg/ml, Beyotime Biotech, China). Images were captured and viewed on a confocal laser scanning microscope (Nikon, Japan) and analyzed with the NIS Elements Viewer Software (Nikon, Japan).

### 2.4 Ussing Chamber experiments

The colonic tissues were dissected and placed in ice-cold Kreb’s buffer containing (in mM, pH 7.2-7.4): 119 NaCl, 4.7 KCl, 25 NaHCO_3_, 1.2 KH_2_PO_4_, 2.5 CaCl_2_, 1.2 MgCl_2_ and 11.1 glucose. The colonic tissues were carefully removed the seromuscular layer in a Sylgard-lined Petri dish. The acquired mucosal tissues were then mounted on the Ussing Chamber systems (VCC MC6; World Precision Instruments, United States), with an exposed area of 0.5 cm^2^. Both sides were bathed with 5 mL Kreb’s solution at 37°C, and gassed with 95% O_2_ and 5% CO_2_. The spontaneous potential difference (PD) was monitored via agar-salt bridges connecting to calomel electrodes, and the appropriate Isc was adjusted via Ag-AgCl electrodes to maintain a zero PD through an automatic voltage clamp. The transepithelial resistance (TER) was calculated from the spontaneous PD and Isc by means of Ohm’s law. After an approximately 30-min equilibration period, the baseline PD, Isc and TER was recorded followed by interventions. To determine the mucosal viability, tissues were stimulated with bethanechol.

### 2.5 Spontaneous and induced epithelial Cl^−^ secretion

To investigate the spontaneous Cl^−^ secretion, the non-selective Cl^−^ channel blockers, glibenclamide (1 mmol/L) and 1,1-dimethyl piperidinium chloride (DPC, 1 mmol/L), the selectively Ca^2+^-dependent Cl^−^ channel blockers, niflumic acid (NFA, 100 μmol/L), 4,4’-diisothiocyanato-stilbene-2,2’-disulfonic acid (DIDS, 250 μmol/L) and 5-nitro-2-(3-phenylpropylamino) benzoic acid (CaCC_inh_-A01, 10 μmol/L), and the selectively cAMP-dependent Cl^−^ channel blocker, CFTR_inh_-172 (10 μmol/L), were added into mucosa-side bath solution respectively. The Cl^−^ channel blocker-induced decrease in Isc (ΔIsc) was considered as the component of spontaneous Cl^−^ secretion. Moreover, 1 μmol/L bethanechol and 1 μmol/L forskolin were added into the serosa-side bath solution respectively to evaluate the epithelial secretory response to secretagogues.

### 2.6 Neural activated Cl^−^ secretion

The mucosal preparation containing intact enteric nerve fibers was used for the assessment of neural activated Cl^−^ secretion. Tetrodotoxin (TTX, 1 μmol/L), a neural blocker, was added to the serosa-side bath solution to assess the never-mediated basal secretion tone. Nerve-activated secretion was stimulated by veratridine (10 μmol/L), a neural activator, and electrical field stimulation (EFS). EFS was applied between two aluminum foil electrodes on the submucosal surface of the tissue to activate the submucosal nerves including secretomotor neurons. The stimulations were rectangular pulses with strength of 5 mA and duration of 0.5 m at 2Hz, 4Hz, 8Hz, 16Hz, 32Hz or 64 Hz applied for a total of 90 s through an adjustable multi-channel electrical stimulator (RM6240, Chengdu Instrument Factory, China). The adjacent EFS carried out at a 15-min-interval. In order to further confirm the cholinergic and VIPergic functions, the tissues were preincubated with the muscarinic receptor antagonist scopolamine (10 μmol/L), the nicotinic receptor antagonist hexamethonium (100 μmol/L) or the specific VPAC receptor antagonist VIP_6-28_ (1 μmol/L) at least 15 min prior to veratridine exposure or EFS treatment.

### 2.7 Drugs and solutions

Except for CFTR_inh_-172 (R&D Systems Inc., MN, United States) and VIP6-28 (American Peptides, Sunnyvale, CA), all drugs were acquired from Sigma-Aldrich Co., LLC, United States. The NFA, DIDS, CaCC_inh_-A01, CFTR_inh_-172, glibenclamide, veratridine and forskolin were dissolved in dimethyl sulphoxide (DMSO), others were prepared in double distilled water. The volume of each chemical added to the chamber did not exceed 10 μL (0.2% of bathing solutions).

### 2.8 Data analysis and statistics

The data were presented as mean ± standard deviation. The Student’s t-test or nonparametric Mann-Whitney test was used for comparisons between groups accordingly. For the comparisons of nerve blocking induced ΔΔIsc between aged and young subjects, a two-way repeated ANOVA with Bonferroni *post hoc* analysis was conducted. The P value <0.05 was considered statistically significant.

## 3 Results

### 3.1 Effects of aging on the histology and baseline secretion of colonic mucosa

**FIGURE 1:**
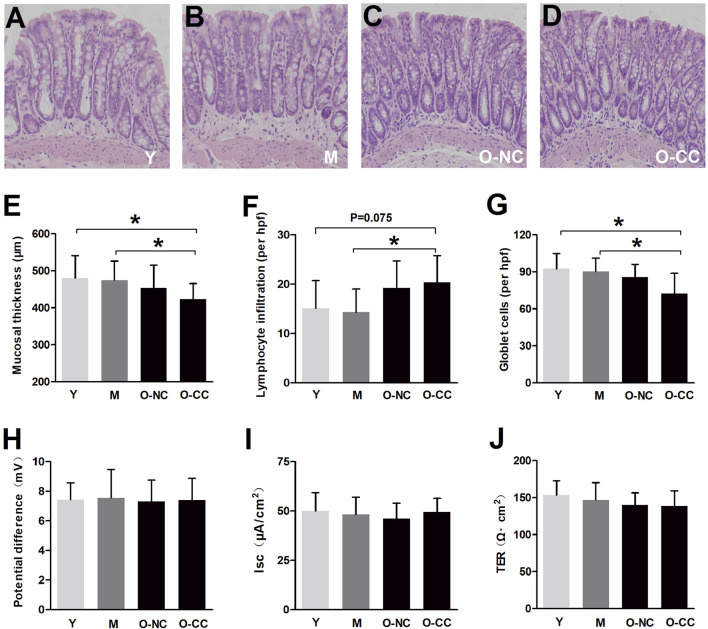
Effects of aging on the histology and baseline secretion of colonic mucosa. **(A-D)** Typical colonic mucosal morphology under HE staining. **(E)** The thickness of mucosal layer. **(F)** The lymphocyte infiltration in the colonic mucosa (per hpf). **(G)** The goblet cell counts in the colonic mucosa (per hpf). The baseline **(H)** short circuit current (Isc), **(I)** potential difference (PD) and **(J)** transepithelial resistance (TER) of the colonic epithelium. Data are displayed as the mean ± SD. N = 8-10; ^*^P < 0.05.

Histological analysis revealed no significant differences in the colonic mucosa among the four groups (Figures 1A–D). Parameters such as mucosal layer thickness, lymphocyte infiltration, and goblet cell counts were not significantly altered with aging. Interestingly, despite the presence of constipation, there were no notable variations in these parameters between the O-NC and O-CC groups. However, it was observed that the O-CC group displayed a decrease in mucosal thickness, an increase in lymphocyte infiltration, and a reduction in goblet cell count compared to the Y/M group (Figures 1E–G). Additionally, the baseline potential difference, baseline secretion levels, and transepithelial resistance (TER) were not affected by aging (Figures 1H–J).

### 3.2 Effects of aging on spontaneous and induced epithelial Cl^−^ secretion

**FIGURE 2:**
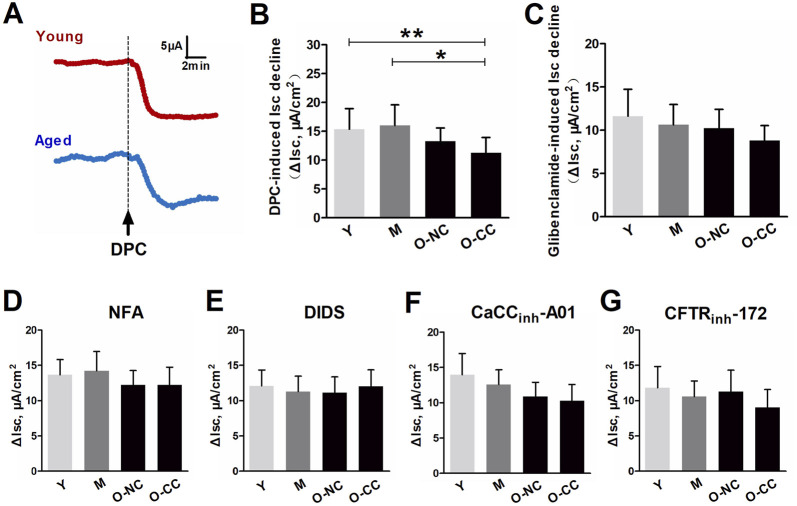
Effects of aging on spontaneous epithelial Cl^−^ secretion. **(A)** Chloride blocking induced obvious decrease in Isc (ΔIsc). **(B, C)** The Isc decrease induced by non-selective Cl^−^ channel blocker DPC and glibenclamide. **(D–F)** The Isc decrease induced by selectively Ca^2+^-dependent Cl^−^ blockers, including NFA, DIDS and CaCCinh-A01. **(G)** The Isc decrease induced by selectively cAMP-dependent Cl^−^ blocking by CFTRinh-172. Data are displayed as the mean ± SD. N = 8-10; ^*^P < 0.05.

Blockage of Cl^−^ channels led to a notable reduction in short-circuit current (ΔIsc), associated with spontaneous Cl^−^ secretion. When comparing the young and elderly subjects, the ΔIsc induced by the non-selective Cl^−^ channel blocker DPC was significantly less in the aged colon, particularly in the O-CC group (Figures 2A, B). In contrast, there was only a downward trend in ΔIsc induced by glibenclamide (Figure 2C). Age or constipation did not lead to significant reductions in ΔIsc induced by selective Ca^2+^-dependent Cl^−^ channel blockers such as NFA, DIDS, and CaCC_inh_-A01 (Figures 2D–F), as well as the selective cAMP-dependent Cl^−^ blocker CFTR_inh_-172 (Figure 2G).

**FIGURE 3:**
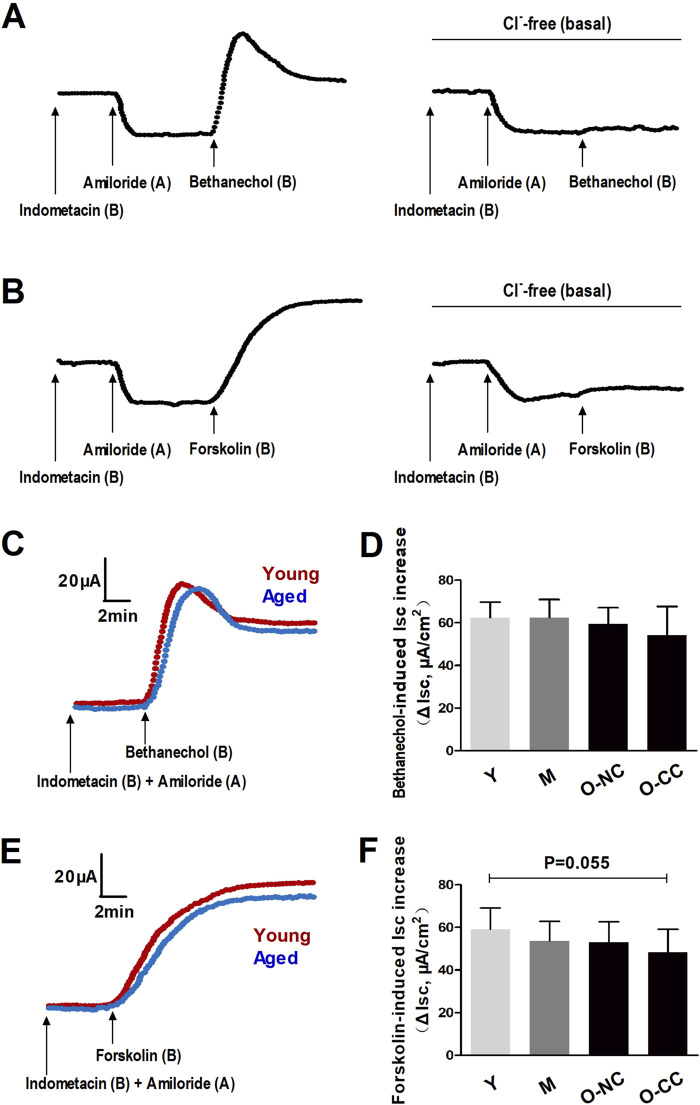
Effects of aging on bethanechol- and forskolin-induced epithelial Cl^−^ secretion. Bethanechol **(A)** and forskolin **(B)** evoked significant increase in Isc (ΔIsc) under epithelial Na^+^ channel (ENaC) blocking with amiloride, which was markedly inhibited in Cl^−^-free condition. **(C, D)** Bethanechol-induced Isc increase in the young and the aged colon. **(E, F)** Forskolin-induced Isc increase in the young and the aged colon. Data are displayed as the mean ± SD. N = 8-10; ^*^P < 0.05.

The ΔIsc induced by bethanechol and forskolin primarily indicated epithelial Cl^−^ outflow, reflecting Ca^2+^-dependent and cAMP-dependent Cl^−^ efflux, respectively. In a Cl^−^-free condition, the increases in Isc induced by bethanechol and forskolin after ENaC blockade with amiloride were significantly inhibited (Figures 3A, B). Additionally, the ΔIsc induced by bethanechol and forskolin remained unaffected by aging or constipation (Figures 3C–F).

### 3.3 Effects of aging on neurogenic Cl^−^ secretion

**FIGURE 4:**
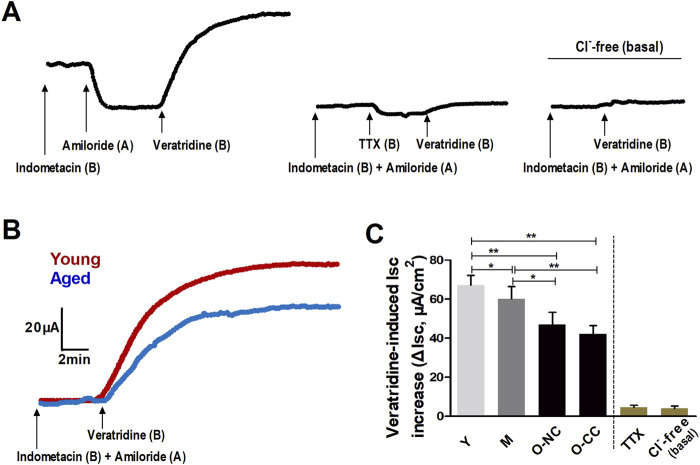
Aging reduced the neurogenic Cl^−^ secretion induced by veratridine administration. **(A)** The neural activator veratridine induced remarkable increase in Isc (ΔIsc), which was almost entirely blocked by TTX, as well as in Cl^−^-free condition. **(B)** Typical curves of veratridine-induced Isc increase in the young and the aged colon. **(C)** The veratridine-induced ΔIsc was declined with aging, and more apparently impaired in the aged with constipation. Data are displayed as the mean ± SD. N = 8-10; ^*^P < 0.05, ^**^P < 0.01.

The neural agonist veratridine led to a notable rise in Isc, which was almost entirely inhibited by the application of tetrodotoxin (TTX) as well as Cl^−^-free condition (Figure 4A). These responses indicated that neural activation prompted a significant outflow of Cl^−^ in colonic mucosa. In elderly subjects, there was a decrease in neurogenic Cl^−^ outflow compared to the young subjects (Figure 4B). Moreover, the ΔIsc induced by veratridine declined with aging, particularly in O-CC group (Figure 4C).

**FIGURE 5:**
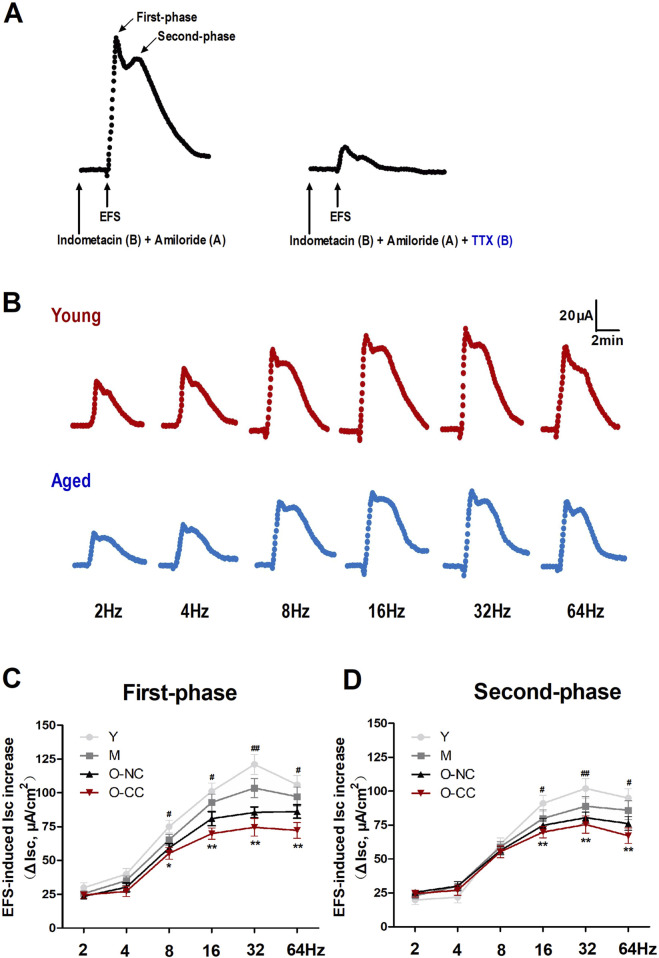
Aging declined the nerve-evoked Cl^−^ secretion induced by EFS treatment. **(A)** EFS activated characteristic biphasic responses in the colonic mucosa, the first-phase and the second-phase response, which were markedly suppressed by neural blocking with TTX. **(B)** EFS stimulated Cl^−^ secretion in a frequency-dependent manner in the young and the aged colon. **(C, D)** The influence of aging on EFS-induced first-phase and second-phase responses in human colon. Data are displayed as the mean ± SD. N = 8-10; ^*^P < 0.05, ^**^P < 0.01 Y vs. O-NC; ^#^P < 0.05, ^##^P < 0.01 Y vs. O-CC.

Furthermore, we evaluated neurogenic Cl^−^ secretion via electrical field stimulation (EFS) method in colonic mucosa. EFS elicited characteristic biphasic responses, comprising a first-phase and a second-phase, both of which were significantly suppressed by TTX administration (Figure 5A). Moreover, EFS elicited Cl^−^ secretion in a frequency-dependent manner, exhibiting maximal effects at frequencies of 16 Hz and 32 Hz. The Cl^−^ secretion evoked by EFS was also weakened in the elderly subjects compared to young subjects (Figure 5B). The responses from both the first and second phases tended to attenuate with aging, particularly in O-CC group (Figures 5C, D).

### 3.4 Effects of cholinergic and VIPergic inhibition on neurogenic Cl^−^ secretion

**FIGURE 6:**
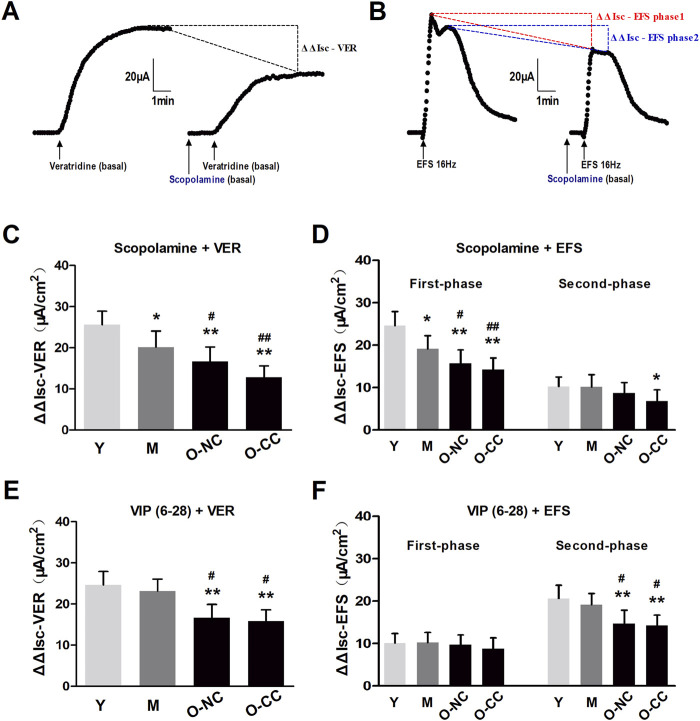
Effects of cholinergic and VIPergic blocking on neurogenic Cl^−^ secretion. **(A)** Effects of scopolamine on Isc increasing (ΔIsc) induced by veratridine exposure (ΔΔIsc-VER). **(B)** Effects of scopolamine on Isc increasing (ΔIsc) induced by EFS, both the first-phase response (ΔΔIsc-EFS phase1) and the second-phase response (ΔΔIsc-EFS phase2). **(C, D)** Scopolamine-induced ΔΔIsc-VER and ΔΔIsc-EFS phase1 were markedly declined with aging, and more apparent in the aged with constipation. **(E, F)** VIP6–28-induced ΔΔIsc-VER and ΔΔIsc-EFS phase2 were markedly decreased in the aged with or without constipation. Data are displayed as the mean ± SD. N = 8-10; ^*^P < 0.05, ^**^P < 0.01 compared with Y group; ^#^P < 0.05, ^##^P < 0.01 compared with M group.

Next, we investigated the effects of the two key secretion-promoting nerves, cholinergic and VIPergic nerve, on the neurogenic Cl^−^ secretion evaluated through difference in ΔIsc (ΔΔIsc) induced by corresponding antagonist after nerve activation in colonic mucosa. Cholinergic blocking by scopolamine treatment decreased the ΔIsc evoked by veratridine exposure (Figure 6A). It also weakened the first-phase and second-phase responses evoked by EFS treatment (Figure 6B). Additionally, scopolamine treatment significantly reduced the ΔΔIsc in elderly individuals, regardless of constipation (Figures 6C, D). The similar pattern was observed with VIPergic inhibition through VIP_6-28_ treatment, which also showed a reduction in ΔΔIsc with aging, specifically in the second-phase response among the elderly (Figures 6E, F).

### 3.5 Characteristics of enteric nerve fibers in aged colonic mucosa

**FIGURE 7:**
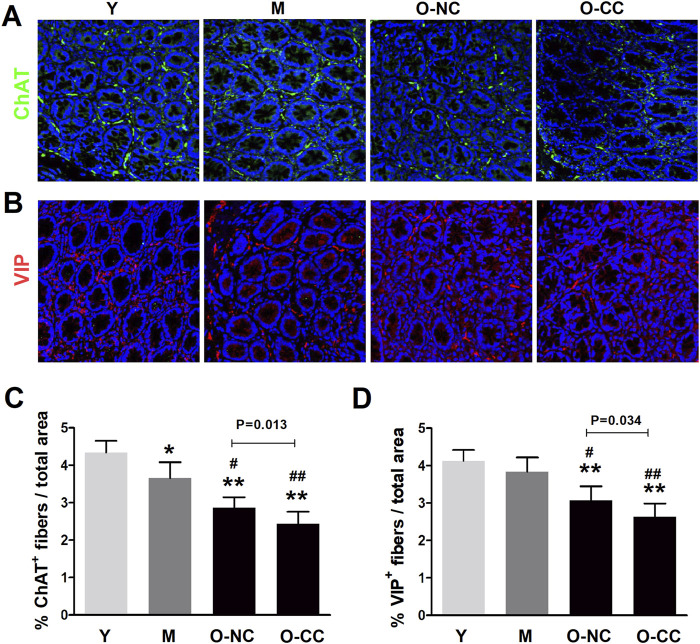
Alterations of enteric nerve fibers in aged colonic mucosa. **(A, B)** Typical images of the distribution of mucosal cholinergic fibers (ChAT^+^, green) and VIPergic fibers (VIP^+^, red) in the young and the aged colon. **(C)** Evident reduction in the density of cholinergic fibers in the aged colonic mucosa. **(D)** More sparse distribution of mucosal VIPergic fibers in aged colon. Data are displayed as the mean ± SD. N = 8-10; ^*^P < 0.05, ^**^P < 0.01 compared with Y group; ^#^P < 0.05, ^##^P < 0.01 compared with M group.

The density of nerve fibers in colonic mucosa were further evaluated. A significant decrease in the density of cholinergic fibers (ChAT^+^) was identified in the elderly subjects (Figures 7A, C). This reduction was similarly observed in the O-CC group compared to the O-NC group (Figures 7A, C). Moreover, there was a noticeable scarcity in the distribution of VIPergic fibers (VIP^+^) among elderly subjects within colonic mucosa, and a similar significant decrease was noted in the O-CC group compared to the O-NC group (Figures 7B, D).

## 4 Discussion

The current study investigated the impact of aging on epithelial secretion in the human colon. It indicated that aging disrupted epithelial Cl^−^ secretion evoked by nerve activity, independent from Ca^2+^-dependent or cAMP-dependent mechanisms. Therefore, the weakness in colonic secretion with aging seemed to be neurogenic, specifically involving cholinergic and VIPergic nerves in the mucosa.

Although the exact causes of aging-related colon disorder were not fully understood, they likely involved epithelial dysfunction. A common observation in aged colon included compromised epithelial barrier integrity, weakened mucosal defense, decreased stem cell renewal, abnormalities in Paneth and goblet cells (Man et al., 2014), and inflammatory infiltration (Sato et al., 2015). Consistently, our results demonstrated a significant decrease in mucus-producing goblet cells and an increase in lymphocyte infiltration in the aged colons. Overall, these results supported a substantial impact of aging on the colonic epithelium and mucosa function, particularly concerning their ability to secrete ions and fluids.

It should be noted that the Isc (short-circuit current) method was commonly used to measure the net electrogenic transport of ions across epithelial tissues, which could be a complex process (Zhang et al., 2007). A decrease in Isc may suggest increased absorption or decreased secretion of anions (such as Cl^−^) and increased cation secretion like potassium (such as K^+^). Conversely, an increase in Isc was usually associated with anion secretion (such as Cl^−^ or HCO_3_^−^) or cation absorption (such as Na^+^) (Zhang et al., 2007). The combination of anions and/or cations transport collectively defined the basal Isc. Our study revealed a slight decrease in ΔIsc by blocking Cl^−^ channels in the aged colon, with a significant reduction in elderly subjects with constipation, indicating impaired spontaneous Cl^−^ secretion in elderly individuals with constipation. Blocking Ca^2+^-dependent and cAMP-dependent pathways did not significantly affect ΔIsc in the aged colon, suggesting that CaCC and CFTR activities remained independent of aging.

Acetylcholine elevated intracellular Ca^2+^ level to stimulates Cl^−^ outflow via CaCC, while forskolin enhanced cAMP level to facilitate Cl^−^ outflow via CFTR (Yeh et al., 2019; Lomasney et al., 2014). Administering bethanechol or forskolin led to an increase in Isc, with no significant differences observed between the colons of young and elderly subject, despite potential changes in muscarinic receptor expression, calcium mobilization, and cAMP response related to aging (Lopes et al., 2006; Hoffmann et al., 2016). These findings supported the absence of significant disruptions in Ca^2+^-dependent and cAMP-dependent Cl^−^ secretion in the aged colon. Aging had been reported to have negative impacts on the function of myenteric and submucosal plexuses, potentially influencing mucosal secretion (Baidoo et al., 2023). Our study explored nerve-evoked epithelial secretion via veratridine exposure whose effects could be almost entirely blocked by TTX (Cavin et al., 2020). Interestingly, the neurogenic secretion-promoting impact seemed to diminish with aging, as the rise in Isc evoked by veratridine treatment tended to decrease in elderly individuals. Different from veratridine-evoked responses, the response initiated by EFS displayed a rising phase followed by a sustained phase, both of which could be fully blocked by TTX (Baldassano et al., 2012; Hirota and McKay, 2006; Baldassano et al., 2009). Our results revealed that the Isc rise evoked by EFS was hindered in the aged colon in a frequency-dependent manner, with varying frequencies of EFS leading to diverse transmitter release patterns (De Schepper et al., 2006; Sanders and Mutafova-Yambolieva, 2021).

EFS primarily targeted submucosal secretomotor neurons and/or excitatory interneurons upstream (Baldassano et al., 2012; Hirota and McKay, 2006; Baldassano et al., 2009). Submucosal secretomotor neurons released ACh and VIP, serving as the key neurotransmitters that contributed to the Isc rises evoked by EFS (Baldassano et al., 2012; Traserra et al., 2023; Baldassano et al., 2009). Specifically, the first-phase response of EFS was predominantly influenced by cholinergic activity, while the second-phase response may involve a VIPergic element (Baldassano et al., 2009). Furthermore, the EFS-induced rise in Isc depended on epithelial Cl^−^ and HCO_3_^−^ secretion, without being reliant on amiloride-sensitive sodium (Na^+^) absorption or the inhibition of receptors for adenosine A1 and A2, purinergic P2X and P2Y, or tachykinin NK1 and NK2 (Krueger et al., 2016). Subsequently, we blocked the cholinergic and VIPergic activities during EFS, and observed a reduction in ΔΔIsc in the aged colon, aligning with our previous hypothesis that neurogenic Cl^−^ secretion diminished with aging, possibly due to cholinergic and VIPergic nerve dysfunction.

Recent findings suggested a substantial decline of approximately 40%–60% in the total neuron count within the aged colon of guinea pigs and rats (Garrido-Gil et al., 2018). Studies proposed that reduced secretion of neurotrophic factors (Becker et al., 2018; Hall, 2002) and an imbalanced ratio between oxygen radical production and antioxidant defense mechanisms (Wade and Cowen, 2004) may contribute to the heightened vulnerability of neurons during the aging process. It was reported that aging reduced the nerve fiber density of HuC/D^+^ subgroup and ChAT^+^ subgroup in myenteric plexus, whereas had little effect on the nNOS-immunopositive nerve fiber density (Nguyen et al., 2022). Nevertheless, the consensus regarding neuron quantities in the submucosal plexus among the elderly remained controversial (Hanani et al., 2004; Budnik et al., 2020; Szymaszkiewicz et al., 2021; Phillips et al., 2007). Our research delved into the examination of ChAT^+^ and VIP^+^ fibers within the colonic mucosa of young and aged individuals, and observed a significant decrease in both fibers as a consequence of aging. Those results reinforced the hypothesis that age-related alterations led to disruptions in epithelial Cl^−^ secretion mediated by cholinergic and VIPergic nerves.

## 5 Conclusion

In summary, this paper reported an age-related impairment of neurogenic Cl^−^ secretion in colonic mucosa, particularly in the elderly suffering from constipation. The diminished function of cholinergic and VIPergic nerves was likely to contribute to the impaired neurogenic secretion in aged colon. Focusing on enteric neurotherapy seemed to be necessary. This paper may assist in understanding the pathophysiology of the elderly constipation.

## Data Availability

The original contributions presented in the study are included in the article/supplementary material, further inquiries can be directed to the corresponding author.

## Abbreviations

CFTR, cystic fibrosis transmembrane regulator; CaCC, Ca^2+^-activated chloride channel; ENaC, epithelial Na^+^ channel; cAMP, cyclic adenosine monophosphate; Fsk, forskolin; ACh, acetylcholine; VIP, vasoactive intestinal polypeptide; ChAT, choline acetyltransferase; PD, potential difference; TER, transepithelial resistance; Isc, short circuit current; DPC, 1,1-dimethyl piperidinium chloride; NFA, niflumic acid; DIDS, 4,4’-diisothiocyanato-stilbene-2,2’-disulfonic acid; TTX, tetrodotoxin; EFS, electrical field stimulation; Y, young; M, middle aged; O-NC, old without constipation; O-CC, old patients with constipation.
